# Cerebral small vessel disease mediates the association between homocysteine and cognitive function

**DOI:** 10.3389/fnagi.2022.868777

**Published:** 2022-07-15

**Authors:** Zhenjie Teng, Jing Feng, Ronghui Liu, Yifan Ji, Jing Xu, Xin Jiang, Huifang Chen, Yanhong Dong, Nan Meng, Yining Xiao, Xiaohua Xie, Peiyuan Lv

**Affiliations:** ^1^Department of Neurology, Hebei Medical University, Shijiazhuang, China; ^2^Department of Neurology, Hebei General Hospital, Shijiazhuang, China; ^3^Department of Endocrinology, Hebei General Hospital, Shijiazhuang, China; ^4^Department of Radiology, Hebei General Hospital, Shijiazhuang, China

**Keywords:** homocysteine, cerebral small vessel disease, cognitive impairment, white matter hyperintensity, cerebral microbleed, lacune, perivascular space

## Abstract

**Objective:**

To investigate the relations of serum total homocysteine (tHcy) with cerebral small vessel disease (CSVD) and cognitive function and evaluate whether CSVD mediates the effect of serum tHcy on cognitive function.

**Methods:**

A total of 1,033 consecutive eligible participants who received serum tHcy, brain magnetic resonance imaging (MRI), and neuropsychological assessment were included in this retrospective study. White matter hyperintensity, lacune, cerebral microbleed, and enlarged perivascular space were evaluated based on brain MRI. We used multivariate binary logistic regression analysis, multivariate ordinal logistic regression analysis, and mediation analyses to assess the relations of serum tHcy with CSVD and cognitive function.

**Results:**

Serum tHcy levels were higher in patients with cognitive impairment than those with no cognitive impairment. Logistic regression analyses showed elevated serum tHcy was associated with cognitive impairment [odds ratio (OR): 10.475; 95% confidence interval (CI): 4.522 to 24.264; *p* < 0.001] and a higher CSVD burden score (OR: 17.151; 95% CI: 8.785 to 33.921; *p* < 0.001) after adjusting potential confounders. Compared with the lowest tHcy quartile, the multivariable-adjusted OR of the highest quartile was 4.851 (95% CI: 3.152 to 7.466; *p* for the trend < 0.001) for cognitive impairment, 3.862 (95% CI: 2.467 to 6.047; *p* for the trend < 0.001) for a severe CSVD burden score. Mediation analyses showed significant moderating effects (9.3–23.6%) by different imaging markers of CSVD on the association between higher serum tHcy levels and cognitive impairment.

**Conclusion:**

Elevated serum tHcy is associated with cognitive impairment and the development of CSVD. A proportion of the association between elevated serum tHcy and cognitive impairment may be attributed to the presence of different imaging markers of CSVD, especially the severe CSVD burden score.

## Introduction

Protecting and promoting brain health have become one of the top global priorities of health policies. With the increases in population aging and life expectancies, cognitive impairment has emerged as one of the leading culprits for poor brain health and imposed a tremendous burden on global public health (Gardener et al., 2015; Wang et al., 2020; Avan et al., 2021).

Cerebral small vessel disease (CSVD) is a prominent determinant of cognitive impairment in the elderly and responsible for about 50% of dementia worldwide (Wardlaw et al., 2019; Hamilton et al., 2021). CSVD is a dynamic whole-brain disorder (Ter Telgte et al., 2018; Wardlaw et al., 2019). Visible imaging markers of the disease on conventional magnetic resonance imaging (MRI) include white matter hyperintensity (WMH), lacune, cerebral microbleed (CMB), and enlarged perivascular space (EPVS) (Wardlaw et al., 2013; Ter Telgte et al., 2018). The total CSVD burden score, combined with these markers into one score, can be more representative of the severity of CSVD (Staals et al., 2014). Although pathological mechanisms of CSVD are incompletely understood, endothelial dysfunction seems to be a pivotal factor (Rajani et al., 2018; Quick et al., 2021). Other factors, such as blood-brain barrier (BBB) dysfunction, hypoperfusion, oxidative stress, and inflammation, also contributed to mechanisms of CSVD (Ihara and Yamamoto, 2016; Grochowski et al., 2018; Low et al., 2019; Wardlaw et al., 2019). Similarly, all factors mentioned above are potential mechanisms underlying cognitive impairment (Nation et al., 2019; Zlokovic et al., 2020).

Homocysteine (Hcy) is one of metabolically related sulfur-containing amino acids (Jakubowski, 2019). Elevated serum total Hcy (tHcy) has been shown to be associated with many diseases, syndromes or outcomes, notably some age-associated disorders, such as cognitive impairment and CSVD (Ostrakhovitch and Tabibzadeh, 2019; Smith and Refsum, 2021). Interestingly, similar to mechanisms of CSVD, possible mechanisms proposed to explain the association between elevated serum tHcy and age-associated disorders include endothelial dysfunction, BBB dysfunction, oxidative stress, and neuroinflammation, although the relationships among these mechanisms are complex and not yet fully understood (Smith and Refsum, 2016; Ostrakhovitch and Tabibzadeh, 2019; Moretti et al., 2021; Sharma et al., 2021). Therefore, there should be some underlying correlation or relationship among those three. However, few studies have explored simultaneously the relationship of serum tHcy with CSVD and cognitive function.

In this study, we aimed to investigate the relationship of serum tHcy with CSVD and cognitive function and to determine whether CSVD (i.e., a total CSVD burden score, WMH, CMB, lacune, EPVS) mediates the effect of serum tHcy on cognitive function.

## Materials and methods

### Participants

This is a retrospective study of patients from the memory clinic in Hebei General Hospital. From September 2016 to October 2020, a total of 1,442 patients were included. Inclusion criteria: (1) aged 50 years and older; (2) completed MRI with adequate sequences for the assessment of CSVD; and (3) completed cognitive function assessment. We excluded those with a symptomatic vascular event within 3 months (*n* = 203), severe neurologic deficit (*n* = 134), and other conditions that may have an impact on cognitive function (*n* = 72), such as epilepsy, malignancy, brain injuries, anxiety, depression, hyperthyroidism, hypothyroidism or carbon monoxide poisoning. Finally, we included 1,033 eligible patients in the analyses.

### Clinical assessment

Following clinical factors per patient were collected in our study: demographic characteristics (age, gender, years of education, height, weight), medical history (hypertension, diabetes, coronary heart disease, stroke), cigarette smoking, and alcohol-drinking status. Laboratory examination results, including total cholesterol (TC), triglyceride (TG), low-density lipoprotein cholesterol (LDL-C), high-density lipoprotein cholesterol (HDL-C), very low-density lipoprotein cholesterol (VLDL-C), uric acid, and serum tHcy, were evaluated after 8 h of overnight fasting. Serum tHcy levels were determined by the enzyme circulation method.

### Brain magnetic resonance imaging data acquisition and evaluation

Magnetic resonance imaging examination was performed in all patients with 3 T magnetic resonance scanners (Signa, GE Healthcare, United States). The complete neuroimaging sequences included T1-weighted imaging (T1WI), T2-weighted imaging (T2WI), fluid-attenuated inversion recovery (FLAIR), diffusion-weighted imaging (DWI), and susceptibility weighted imaging (SWI). MR scanning parameters: T1WI: repetition time (TR)/echo time (TE) = 1,909/20.2 milliseconds (ms), slice thickness = 5 millimeters (mm); T2WI: TR/TE = 5,000/125 ms, slice thickness = 5 mm; FLAIR: TR/TE = 8,502/159.4 ms, slice thickness = 5 mm; DWI: TR/TE = 4,800/81.7 ms, slice thickness = 5 mm and SWI: TR/TE = 78.6/47.6 ms, slice thickness = 2 mm.

Imaging markers of CSVD (WMH, CMB, lacune, EPVS) were evaluated independently by two readers (YJ and JX) according to the standard published criteria (Wardlaw et al., 2013). In case of disagreement on any markers, a radiologist (RL) assessed the images in order to achieve consensus. All ratings were performed blinded to all patient data. WMH was displayed as hyperintensity on T2WI and FLAIR, without cavitation. The severity of periventricular WMH (pWMH) (range, 0–3) and the deep WMH (dWMH) (range, 0–3) was evaluated using Fazekas rating scale (Fazekas et al., 1993). A lacune was classified as a round or ovoid, subcortical cavity, between 3 and 15 mm in diameter, with a similar signal of cerebrospinal fluid on T1WI or T2WI, following the territory of a perforating arteriole (Wardlaw et al., 2013). CMB was a small (2–10 mm in diameter) area of signal void on SWI and graded using the Microbleed Anatomical Rating Scale (Gregoire et al., 2009). The presence, number, and location (lobar or deep) of CMB were recorded. EPVS was defined as a round or linear, cerebrospinal fluid-filled cavity with a diameter generally smaller than 3 mm on all sequences and measured in two different regions: the basal ganglia (BG) and centrum semiovale (CSO). In line with previous studies, EPVS in CSO or BG was rated on a semi-quantitative scale from 0 to 4 (0 = none, 1 = 1–10, 2 = 11–20, 3 = 21–40, and 4 > 40) (Doubal et al., 2010). Moderate to severe EPVS was defined when the score ≥ 2 in the CSO or BG (Javierre-Petit et al., 2020). The total CSVD burden score was rated on an ordinal scale from 0 to 4. A point was awarded for each of the following: severe pWMH (score = 3) or moderate to severe dWMH (score ≥ 2), presence of lacune, any deep CMB, and moderate to severe EPVS in BG (Huijts et al., 2013; Staals et al., 2015; Lau et al., 2017). The severe CSVD burden score was defined when the score > 2 (Kim et al., 2021).

### Neuropsychological assessment

All participants received neuropsychological assessment using the standardized translated version of Mini-Mental State Examination (MMSE), which has been validated for Chinese adults. The education levels are strongly recommended to consider when interpreting the results of the MMSE. Therefore, according to a previously published protocol, the optimal cut-off points for cognitive impairment screening were limited to 17 for illiterate, 20 for individuals with 1–6 years of education, and 24 for individuals with 7 or more years of education (Li et al., 2016).

### Statistical analyses

Continuous variables were expressed as mean (standard deviation) or median (the interquartile range) as appropriate and analyzed by Mann–Whitney *U* tests or *T* tests. Categorical variables were presented with case (percentage) and analyzed by *χ^2^* tests. Ordinal variables, such as the total CSVD burden score, were analyzed by Mann–Whitney *U* tests or Kruskal–Wallis tests based on grouping. Multivariate binary logistic regression was performed to determine whether serum tHcy was a potential risk factor of cognitive impairment. Tests for trend were conducted with the use of quartiles of serum tHcy as a continuous variable by assigning the median values of the quartiles to the variable. The tHcy was transformed to log scale in logistic regression or tests for the trend. The receiver operating characteristic (ROC) curve of serum tHcy levels was drawn, and the optimal cut-off point of serum tHcy levels in patients with cognitive impairment was predicted according to the maximum value of the Youden Index. The participants were dichotomized into a higher tHcy levels group and a lower tHcy levels group by using the optimal cut-off point. Values of *p* < 0.05 were considered statistically significant. All above statistical analyses were performed using SPSS software package 21.0 (IBM corporation, Armonk, NY, United States).

Multivariate ordinal logistic regression was performed in R, version 4.1.0 (R Foundation for Statistical Computing, Vienna, Austria) using MASS Package, version 7.3–54 to investigate the cross-sectional relation between serum tHcy and the total CSVD burden score. Mediation models were used to assess whether different imaging markers of CSVD (i.e., the severe CSVD burden score, severe pWMH or moderate to severe dWMH, lacune, deep or lobar CMB and moderate to severe BG-EPVS or CSO-EPVS) mediate the relation between serum tHcy levels and cognitive impairment by using bruceR and mediation Packages in R. The function can automatically judge the model type and also automatically conduct mean-centering before model building. The number of bootstrap samples in each analysis was set to 5,000 to obtain a more robust estimate of the effect.

## Results

### Participants’ characteristics

This study included 1,033 patients. Mean age of the participants was 66.4 ± 8.9 years, and 51% (*n* = 527) were male. All individuals were divided into a cognitive impairment group (*n* = 368) and a no-cognitive-impairment group (*n* = 665) according to the points of MMSE. Characteristics of the participants between the two groups are presented in Table 1. The patients with cognitive impairment were significantly older, more likely to be men and lower educated than those with no cognitive impairment (*p* < 0.05). The frequencies of hypertension, history of stroke, severe pWMH, moderate to severe dWMH, presence of lacune, presence of deep or lobar CMB, and moderate to severe BG-EPVS were higher in the participants with cognitive impairment (*p* < 0.05). The cognitive impairment group presented higher serum tHcy levels and a total CSVD burden score than the no-cognitive-impairment group (*p* < 0.05).

**TABLE 1:** Characteristics of the study participants between cognitive impairment and no-cognitive-impairment groups.

Variable	Cognitive impairment group (*n* = 368)	No cognitive impairment group (*n* = 665)	*p*-value
Age, mean (SD), year	68.8 ± 8.9	65.1 ± 8.7	<0.001*
Sex (male), n (%)	205 (55.7)	322 (48.4)	0.025*
Education, median (IQR), year	9 (6–12)	11 (6–12)	<0.001*
Body mass index, mean (SD), kg/m^2^	24.7 ± 3.2	24.9 ± 3.0	0.177
Current smoking, n (%)	77 (20.6)	109 (16.4)	0.069
Alcohol use, n (%)	53 (14.4)	78 (11.7)	0.216
Hypertension, n (%)	262 (71.2)	376 (56.5)	<0.001*
Diabetes, n (%)	100 (27.2)	264 (24.7)	0.375
Coronary heart disease, n (%)	71 (19.3)	121 (18.2)	0.664
History of stroke	136 (37.0)	177 (26.6)	0.001*
TC, median (IQR), mmol/L	4.52 (3.82–5.34)	4.62 (3.90–5.40)	0.118
TG, median (IQR), mmol/L	1.21 (0.87–1.70)	1.27 (0.90–1.80)	0.373
HDL-C, median (IQR), mmol/L	1.12 (0.95–1.30)	1.16 (0.99–1.35)	0.050
LDL-C, median (IQR), mmol/L	2.92 (2.37–3.56)	3.00 (2.43–3.60)	0.209
VLDL-C, median (IQR), mmol/L	0.43 (0.28–0.60)	0.44 (0.30–0.61)	0.652
Uric acid, median (IQR), μmol/L	295 (239–359)	290 (244–354)	0.751
Serum tHcy^a^, median (IQR), μmol/L	1.24 (1.13–1.36)	1.14 (1.05–1.23)	<0.001*
Total CSVD burden score 0, n (%) 1, n (%) 2, n (%) 3, n (%) 4, n (%)	24 (6.5) 47 (12.8) 65 (17.7) 117 (31.8) 115 (31.3)	184 (27.7) 165 (24.8) 148 (22.2) 101 (15.2) 67 (10.1)	<0.001*
pWMH (score = 3), n (%) dWMH (score ≥ 2), n (%)	169 (45.9) 259 (70.4)	123 (18.5) 261 (39.2)	<0.001* <0.001*
Presence of lacune, n (%)	281 (76.4)	301 (45.3)	<0.001*
Presence of deep CMB, n (%) Presence of lobar CMB, n (%)	166 (45.1) 98 (26.6)	152 (22.9) 129 (19.4)	<0.001* 0.007*
BG-EPVS (score ≥ 2), n (%) CSO-EPVS (score ≥ 2), n (%)	279 (75.8) 271 (73.6)	314 (47.2) 483 (72.6)	<0.001* 0.726

*p < 0.05. SD, standard deviation; IQR, interquartile range; TC, total cholesterol; TG, triglyceride; LDL-C, low-density lipoprotein cholesterol; HDL-C, high-density lipoprotein cholesterol; VLDL-C, very low-density lipoprotein cholesterol; tHcy, total homocysteine; CSVD, cerebral small vessel disease; pWMH, periventricular white matter hyperintensity; dWMH, deep white matter hyperintensity; CMB, cerebral microbleed; BG, basal ganglia; CSO, centrum semiovale; EPVS, enlarged perivascular spaces. ^a^The variable was transformed to the log scale.

### Serum total homocysteine levels and cognitive impairment

**FIGURE 1:**
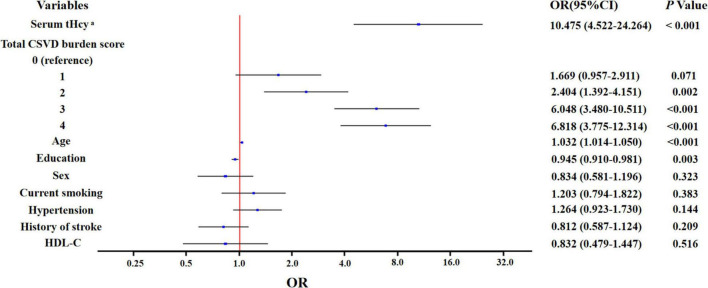
Multivariate binary logistic regression analysis for the associated factors with cognitive impairment. ^a^The variable was transformed to log scale.

In unadjusted binary logistic regression analysis, elevated serum tHcy was associated with cognitive impairment [odds ratio (OR): 27.05; 95% confidence interval (CI): 12.80 to 57.15; *p* < 0.001]. In multivariate binary logistic regression analysis, elevated serum tHcy was independently associated with cognitive impairment (OR: 10.475; 95% CI: 4.522 to 24.264; *p* < 0.001) after further adjustment of potential confounders, such as a total CSVD burden score, age, sex, education, hypertension, history of stroke, and HDL-C (Figure 1).

The levels of serum tHcy were positively associated with the risk of cognitive impairment (Table 2). The OR of cognitive impairment of the highest quartile of serum tHcy levels compared with the lowest was 5.004 (95% CI: 3.267 to 7.665; *p* for the trend < 0.001) after adjusting for age, sex, and education. In addition, the OR was attenuated after further adjustment for hypertension, history of stroke, HDL-C, and current smoking, in which the highest quartile of serum tHcy levels compared with the lowest had an OR of 4.851 (95% CI: 3.152 to 7.466; *p* for the trend < 0.001). Further analyses showed the ORs were attenuated again in a certain extent after controlling for different markers of CSVD except for lobar CMB (i.e., a severe CSVD burden score, severe pWMH or moderate to severe dWMH, presence of lacune, presence of deep CMB, and moderate to severe BG-EPVS or CSO-EPVS).

**TABLE 2:** ORs (and 95% CIs) of cognitive impairment by quartiles of serum tHcy levels^1^.

	Serum tHcy levels^a^				
	
	Quartile 1	Quartile 2	Quartile 3	Quartile 4	*p*-value for trend^2^
Model 1	1.00 (reference)	1.538 (1.005–2.354)	2.335 (1.542–3.534)	5.004 (3.267–7.665)	<0.001
Model 2	1.00 (reference)	1.539 (1.002–2.363)	2.174 (1.430–3.305)	4.851 (3.152–7.466)	<0.001
Model 3	1.00 (reference)	1.490 (0.953–2.329)	1.715 (1.105–2.662)	3.562 (2.260–5.616)	<0.001
Model 4	1.00 (reference)	1.486 (0.961–2.297)	1.962 (1.280–3.006)	4.254 (2.740–6.603)	<0.001
Model 5	1.00 (reference)	1.554 (1.003–2.408)	2.019 (1.319–3.089)	4.533 (2.925–7.024)	<0.001
Model 6	1.00 (reference)	1.438 (0.928–2.229)	1.893 (1.234–2.904)	4.140 (2.666–6.430)	<0.001
Model 7	1.00 (reference)	1.532 (0.997–2.356)	1.941 (1.268–2.971)	4.056 (2.604–6.319)	<0.001
Model 8	1.00 (reference)	1.539 (1.002–2.363)	2.175 (1.427–3.315)	4.855 (3.141–7.504)	<0.001
Model 9	1.00 (reference)	1.494 (0.967–2.310)	1.957 (1.279–2.994)	4.398 (2.837–6.817)	<0.001

*^1^ORs and 95% CIs were calculated with the use of the binary logistic regression model. ^2^Tests for the trend were conducted by treating the quartiles as a continuous variable and assigning the median for each quartile. ^a^The variable was transformed to the log scale. Model 1: Adjusted for age, sex, and education. Model 2: Model 1 plus additional adjustments for hypertension, history of stroke, HDL-C, and current smoking. Model 3: Model 2 plus additional adjustment for the total CSVD burden score. Model 4: Model 2 plus additional adjustment for pWMH (score = 3). Model 5: Model 2 plus additional adjustment for dWMH (score ≥ 2). Model 6: Model 2 plus additional adjustment for presence of lacune. Model 7: Model 2 plus additional adjustment for presence of deep CMB. Model 8: Model 2 plus additional adjustment for presence of lobar CMB. Model 9: Model 2 plus additional adjustment for BG-EPVS (score ≥ 2).*

### Serum total homocysteine levels and cerebral small vessel disease

**FIGURE 2:**
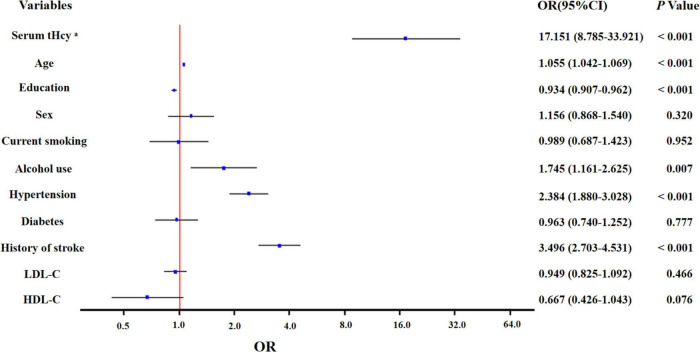
Multivariate ordinal logistic regression analysis for the associated factors with total CSVD burden score. ^a^The variable was transformed to log scale.

In unadjusted ordinal logistic regression analysis, increased serum tHcy was associated with a higher total CSVD burden score (OR: 29.05; 95% CI: 15.67 to 54.62; *p* < 0.001). This trend remained statistically significant (OR: 17.151; 95% CI: 8.785 to 33.921; *p* < 0.001) after adjusting for age, sex, education, current smoking, alcohol use, hypertension, diabetes, history of stroke, LDL-C, and HDL-C (Figure 2).

There was a significant trend among quartiles of serum tHcy levels and risk of the severity of different imaging markers of CSVD, except for CSO-EPVS after controlling for age, sex, education, current smoking, alcohol use, hypertension, diabetes, history of stroke, LDL-C, and HDL-C (Table 3). The multivariable-adjusted ORs of all imaging markers of CSVD were not statistically different for the second quartile of serum tHcy levels (ORs ranging from 0.978 to 1.505), but significantly higher except for CSO-EPVS (ORs ranging from 1.914 to 6.272) for the highest quartile, as compared with the lowest quartile. Interestingly, the multivariate-adjusted OR of deep CMB for the highest quartile of serum tHcy levels compared with the lowest was 6.272 (95% CI: 3.949 to 9.963; *p* for the trend < 0.001), higher than multivariate-adjusted OR of the severe CSVD burden score, which was 3.862 (95% CI: 2.467 to 6.047; *p* for the trend < 0.001).

**TABLE 3:** ORs (and 95% CIs) of different markers of CSVD according to quartiles of serum tHcy levels^1^.

	Serum tHcy levels^a^				
	
Markers of CSVD	Quartile 1	Quartile 2	Quartile 3	Quartile 4	*p*-value for trend^2^
Total CSVD burden (score > 2)	1.00 (reference)	1.115 (0.713–1.745)	2.337 (1.520–3.593)	3.862 (2.467–6.047)	<0.001
pWMH (score = 3)	1.00 (reference)	1.505 (0.915–2.477)	2.600 (1.619–4.176)	3.501 (2.146–5.712)	<0.001
dWMH (score ≥ 2)	1.00 (reference)	0.978 (0.657–1.455)	1.795 (1.203–2.680)	1.914 (1.256–2.918)	<0.001
Presence of lacune	1.00 (reference)	1.316 (0.881–1.966)	2.182 (1.446–3.294)	2.925 (1.883–4.545)	<0.001
Presence of deep CMBs	1.00 (reference)	1.106 (0.677–1.807)	3.357 (2.146–5.253)	6.272 (3.949–9.963)	<0.001
Presence of lobar CMBs	1.00 (reference)	1.223 (0.727–2.056)	2.564 (1.587–4.145)	3.241 (1.985–5.294)	<0.001
BG-EPVS (score ≥ 2)	1.00 (reference)	1.243 (0.850–1.817)	2.079 (1.403–3.080)	2.234 (1.475–3.384)	<0.001
CSO-EPVS (score ≥ 2)	1.00 (reference)	0.988 (0.663–1.474)	0.973 (0.648–1.463)	0.759 (0.496–1.161)	0.183

*^1^ORs and 95% CIs were calculated with the use of the binary logistic regression model adjusted for age, sex, education, current smoking, alcohol use, hypertension, diabetes, history of stroke, LDL-C, and HDL-C. ^2^Tests for the trend were conducted by treating the quartiles as a continuous variable and assigning the median for each quintile. ^a^The variable was transformed to the log scale.*

### Mediation models

**FIGURE 3:**
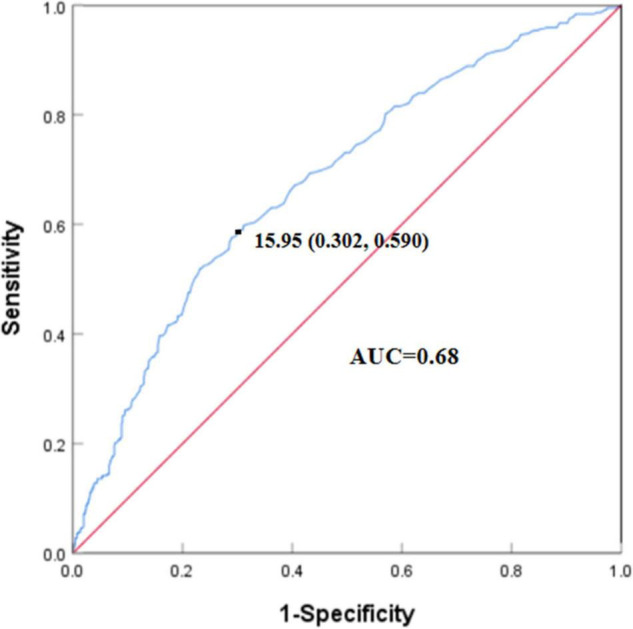
Receiver operating characteristic (ROC) curve of serum tHcy levels for cognitive impairment. The optimal cut-off point of serum tHcy levels of patients with cognitive impairment was 15.95 μmol/L. The specificity was 0.698 (1–0.302) and sensitivity was 0.590. The area under the curve (AUC) was 0.68.

The optimal cut-off point of serum tHcy levels of the patients with cognitive impairment was 15.95 μmol/L, and the area under the curve (AUC) was 0.68 (Figure 3). To further explore the association between serum tHcy and cognitive function, we dichotomized the whole cohort into a higher tHcy levels group and a lower tHcy levels group based on the optimal cut-off point.

**FIGURE 4:**
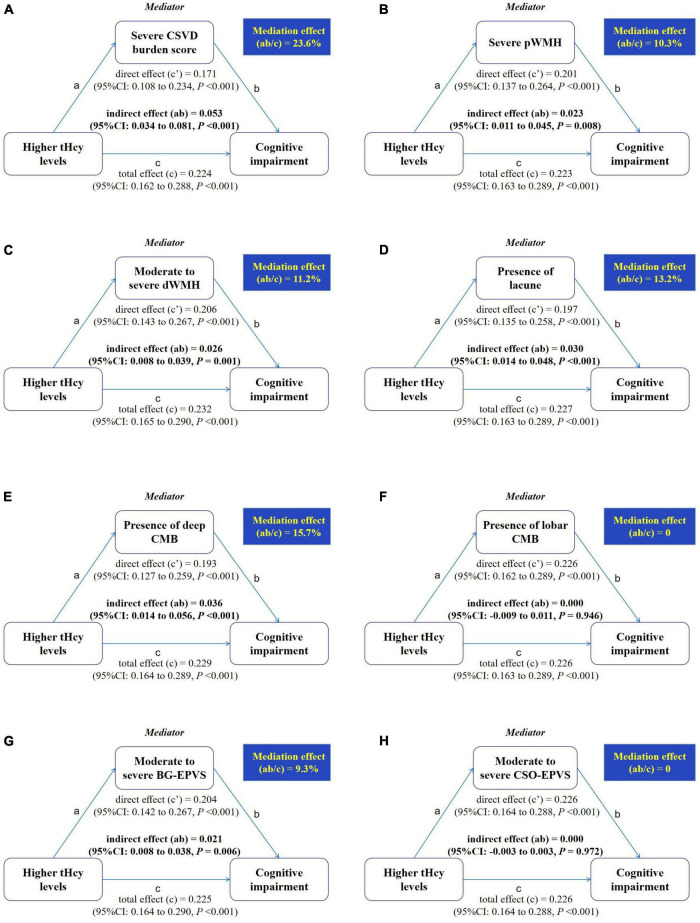
Mediation by different markers of CSVD in the relation between higher serum tHcy levels and cognitive impairment. Mediation analyses are shown for different markers of CSVD as mediators in the relation between higher serum tHcy levels and cognitive impairment. **(A)** The mediator is a severe CSVD burden score. **(B)** The mediator is severe pWMH. **(C)** The mediator is moderate to severe dWMH. **(D)** The mediator is presence of lacune. **(E)** The mediator is presence of deep CMB. **(F)** The mediator is presence of lobar CMB. **(G)** The mediator is moderate to severe BG-EPVS. **(H)** The mediator is moderate to severe CSO-EPVS. The mediation effect is the indirect effect expressed as a percentage of the total effect, that is, the proportion of the relation between the independent and dependent variables attributable to mediation.

Results of the mediation analyses are presented in Figure 4. The direct effect (c′) and total effect (c) were significantly present between higher tHcy levels and cognitive impairment. When the severe CSVD burden score was added to the model, the indirect (i.e., mediating) effect (ab) of the severe CSVD burden score was significant for the relation between higher tHcy levels and cognitive impairment, and 23.6% of the total effect was attributable to mediation by the presence of the severe CSVD burden score after adjusting for age, sex, education, current smoking, alcohol use, hypertension, diabetes, history of stroke, LDL-C, and HDL-C (ab = 0.053; 95% CI:0.034 to 0.081, *p* < 0.001). Similar indirect effects mediated by severe pWMH or moderate to severe dWMH, presence of lacune, presence of deep CMB, and moderate to severe BG-EPVS were observed, although mediation effects (9.3–15.7%) were lower than the severe CSVD burden score (23.6%). In contrast, there was no significant mediation by presence of lobar CMB or moderate to severe CSO-EPVS (*p* > 0.05).

## Discussion

In this observational study, we investigated the associations of serum tHcy with CSVD and cognitive function. We found that elevated serum tHcy was an independent risk factor in cognitive impairment and the development of CSVD. These positive associations tended to be stronger for those with higher serum tHcy levels. Moreover, the presence of the severe CSVD burden score, severe pWMH, moderate to severe dWMH, lacune, deep CMB or moderate to severe BG-EPVS was a significant mediator in the relation between higher tHcy levels and cognitive impairment. Furthermore, although temporal relations cannot be assumed from this cross-sectional study, these findings by mediation models may support the hypothesis that raised serum tHcy may aggravate CSVD, which, in turn, increases the risk of cognitive impairment.

Consistent with the results of previous studies, increased serum tHcy was associated with the development of different markers of CSVD (Kloppenborg et al., 2011; Miwa et al., 2016; Nam et al., 2019). However, to our knowledge, few studies explored the association between serum tHcy and the total CSVD burden score, which reflects the overall burden and is more representative of CSVD (Staals et al., 2014). We observed the levels of serum tHcy were positively associated with the total CSVD burden score, which suggested that increased serum tHcy may accelerate the progression of CSVD. Additionally, we also found stronger associations (ORs ranging from 3.562 to 6.272) between serum tHcy and the severity of different markers of CSVD (except for CSO-EPVS) in the highest quartile of serum tHcy levels, as compared with the lowest quartile of serum tHcy levels, which was similar as the result of previous SMART-MR study (Kloppenborg et al., 2014). Although the specific mechanism between serum tHcy and CSVD remains unclear, several possible mechanisms have been proposed to explain the relationship. Accumulating lines of evidence indicate that endothelial dysfunction plays a crucial role in the relationship between serum tHcy and CSVD (Hassan et al., 2004; Moretti et al., 2021). Elevated tHcy levels can lead to endothelial dysfunction through many processes (Jan et al., 2021). In addition, oxidative stress, inflammation and promoted neurodegenerative processes also contributed to the relationship of serum tHcy and CSVD (Moretti et al., 2021). Of note, we did not observe the relationship between CSO-EPVS or lobar CMB and serum tHcy in this study, which suggested BG-EPVS or deep CMB was specifically related to CSVD compared with CSO-EPVS or lobar CMB. The result was the same as our previous study with small sample sizes (Ji et al., 2020), which may provide further evidence for understanding the different pathologies due to different locations of EPVS or CMB (Vernooij et al., 2008; Wardlaw et al., 2020).

In this study, we found that elevated tHcy was associated with an increased risk of cognitive impairment, as reported previously (Seshadri et al., 2002; Miwa et al., 2016; Di Meco et al., 2019; Zhou and Chen, 2019; Chen et al., 2020). Some possible mechanisms have been proposed to explain the association between elevated serum tHcy levels and cognitive impairment, such as endothelial dysfunction, BBB dysfunction, oxidative stress, and neuroinflammation (Smith and Refsum, 2016; Ostrakhovitch and Tabibzadeh, 2019; Sharma et al., 2021). A population-based prospective cohort study suggested that both high (≥10.6 μmol/L) and low serum tHcy (≤8.9 μmol/L) increased the risk of dementia in older adults (Bae et al., 2021). However, another recent prospective cohort study has found that hazard ratios for dementia were small and non-significant in lower serum tHcy levels, but more than doubled for the higher (≥11.5 μmol/L) (Chen et al., 2020). In this study, we also found that the levels of serum tHcy were associated with the risk of cognitive impairment in a dose-dependent manner. Inconsistent with the above findings, the median and cut-offs of serum tHcy levels were higher in our study, which could be due to older patients, a higher frequency of vascular risk factors and lesions of CSVD. Also because of this, we observed a stronger association (ORs ranging from 3.562 to 5.004) between the highest quartile of serum tHcy levels and cognitive impairment, which was one of the strengths in our research.

Cerebral small vessel disease, which is closely related to both cognitive impairment and serum tHcy, should be taken into account when exploring the association between serum tHcy and cognitive impairment, yet few do. In this study, we observed that magnitude of the association was heterogeneous between serum tHcy and cognitive impairment after taking account of different imaging markers of CSVD, that is, the strengths of associations between serum tHcy and cognitive function decreased when different markers of CSVD besides lobar CMB were included. This finding was in line with previous results in an observational study (Miwa et al., 2016). Nevertheless, that study did not further explore the moderating effect of CSVD between serum tHcy and cognitive function. Another study conducted by Dufouil et al. (2003) indicated that WMH did not mediate the relationship between elevated tHcy levels and cognitive decline in healthy elderly people, although tHcy was an independent risk factor in cognitive impairment. In addition, other markers of CSVD, such as CMB or EPVS, were not discussed in their research. However, in our study, we found different imaging markers of CSVD (i.e., presence of the severe CSVD burden score, severe pWMH, moderate to severe dWMH, lacune, deep CMB, moderate to severe BG-EPVS) had significant moderating effects on the association between increased serum tHcy levels and cognitive impairment, findings not characterized previously. The moderating effects were absent in the lobar CMB and CSO-EPVS, providing further evidence to understand the pathogenesis of different locations of CMB or EPVS. Moreover, our results supported the causal pathway in which elevated serum tHcy aggravated the development of CSVD, which, in turn, increased the risk of cognitive impairment.

Although our research includes some novel findings, some limitations should be acknowledged. First, due to the retrospective design of our study, we were unable to investigate causality. However, results of the mediation analyses support the causal pathway in which increased serum tHcy could promote the development of CSVD, which, in turn, increases the risk of cognitive impairment. Second, as the individuals in our study were recruited from one center in China, the results may not be generalizable to populations with different backgrounds. Third, other potential confounders, such as B vitamin status, which can affect serum tHcy levels or even cognitive function (Smith and Refsum, 2016) and APOE genotype, were not evaluated. In addition, medical history of the participants was not included in this study. They may bias our analyses in the study. Future prospective studies are needed to address this issue. Fourth, demented and non-demented patients were not distinguished in the current study. Future follow-up studies are needed to address this issue. Finally, MMSE, a cognitive screening tool, cannot capture some nuances of specific cognitive domains, such as an executive or visuospatial domain. Therefore, broader neuropsychological testings should be applied in future studies to investigate the relationship between serum tHcy and different cognitive domains.

## Conclusion

Our study provided evidence that serum tHcy was associated with CSVD and cognitive function. We found that elevated serum tHcy was an independent risk factor in cognitive impairment and the development of CSVD. More importantly, different imaging markers of CSVD showed significant moderating effects on the association between higher tHcy levels and cognitive impairment. These results suggested that elevated serum tHcy may lead to cognitive impairment through the presence of different imaging markers of CSVD, especially the severe CSVD burden score. However, to better explore causality of serum tHcy with CSVD and cognitive function, further large prospective studies are warranted.

## Data availability statement

The original contributions presented in this study are included in the article/supplementary material, further inquiries can be directed to the corresponding author.

## Ethics statement

The studies involving human participants were reviewed and approved by the Ethical Committee of Hebei General Hospital. Written informed consent for participation was not required for this study in accordance with the national legislation and the institutional requirements.

## Author contributions

PL contributed to the design of work and revised the manuscript. ZT and JF wrote the manuscript. HC, YD, and NM contributed to acquisition and interpretation of data. RL, YJ, JX, XJ, YX, and XX made substantial contributions to data acquisition and analysis. All authors contributed to the article and approved the submitted version.

## Conflict of interest

The authors declare that the research was conducted in the absence of any commercial or financial relationships that could be construed as a potential conflict of interest.

## Publisher’s note

All claims expressed in this article are solely those of the authors and do not necessarily represent those of their affiliated organizations, or those of the publisher, the editors and the reviewers. Any product that may be evaluated in this article, or claim that may be made by its manufacturer, is not guaranteed or endorsed by the publisher.
